# Novel Side-Chain Type Sulfonated Poly(phenylquinoxaline) Proton Exchange Membranes for Direct Methanol Fuel Cells

**DOI:** 10.3390/membranes12100952

**Published:** 2022-09-28

**Authors:** Dongxia Liang, Qin Wu, Daxin Shi, Yaoyuan Zhang, Hansheng Li, Kangcheng Chen

**Affiliations:** School of Chemistry and Chemical Engineering, Beijing Institute of Technology, Beijing 100081, China

**Keywords:** proton exchange membrane, poly (phenylquinoxaline), post-sulfonation, direct methanol fuel cell

## Abstract

Side-chain type sulfonated poly(phenylquinoxaline) (SPPQ)-based proton exchange membranes (PEMs) with different ionic exchange capacity (IEC) were successfully synthesized by copolymerization from 4,4′-bis (2-diphenyletherethylenedione) diphenyl ether, 4,4′-bis (2-phenylethylenedione) diphenyl ether and 3,3′,4,4′-tetraaminobiphenyl, and post-sulfonation process. The sulfonic acid groups were precisely grafted onto the *p*-position of phenoxy groups in the side chain of PPQ after the convenient condition of the post-sulfonation process, which was confirmed by ^1^H NMR spectra and FTIR. The sulfonic acid groups of side-chain type SPPQ degraded at around 325 °C, and their maximum stress was higher than 47 MPa, indicating great thermal and mechanical stability. The water uptake increased with the increasing IEC and temperature. The size change in their plane direction was shown to be lower than 6%, indicating the stability of membrane electrode assembly. The SPPQ PEMs displayed higher proton conductivity than that of main chain. In the single cell test, the maximum power density of side-chain type SPPQ-5 was 63.8 mW cm^−2^ at 20 wt% methanol solution and O_2_ at 60 °C, which is largely higher than 18.4 mW cm^−2^ of NR212 under the same conditions. The SPPQ PEMs showed high performance (62.8 mW cm^−2^) even when the methanol concentration was as high as 30 wt%.

## 1. Introduction

The direct methanol fuel cell (DMFC) is a classic piece of equipment in a proton exchange membrane fuel cell (PEMFC), which has drawn much attention as a clean and efficient energy source [1]. The proton exchange membrane (PEM), one of the key components in the PEMFC, plays an essential role in conducting protons and most importantly to isolate the crossover of methanol between the anode and cathode [2,3]. The PEM determines the performance and operation temperature in a DMFC system [4]. Therefore, PEMs should have the characteristics of low fuel permeability, reasonable high proton conductivity and good physicochemical stability. The widely used perfluorosulfonic acid polymers (PFSAs) as materials for PEM, such as Nafion, exhibit excellent proton conductivity and electrochemical stability [5]. However, their disadvantages, such as high methanol crossover, low operation cell temperature, etc., seriously limit their further applications [6]. Sulfonated aromatic polymers have the advantages of excellent thermal stability and mechanical properties, as well as low cost, making it possible for them to replace PFSAs as PEMs [7,8,9,10,11].

Poly(phenylquinoxaline)s (PPQs), a kind of thermoplastic polymer, were developed early and are famous for their superior chemical and thermal stability, mechanical properties, and good processability [12]. Sulfonated poly(phenylquinoxaline)s (SPPQs) polymer is obtained by grafting sulfonic acid groups onto PPQs by sulfonation reaction. SPPQs not only inherit the excellent properties brought by their rigid main chain structure but also have sulfonic groups for conducting protons [13,14]. It has been reported that SPPQs were used to prepare proton exchange membranes [15,16,17,18,19,20,21,22].

Zhang et al. [20] first synthesized a series of SPPQ polymers from a sulfonated tetraamine and aromatic bis-benzils through the direct copolymerization method, and the proton conductivity of PEMs-based SPPQ was high. Nevertheless, direct copolymerization methods are limited as a result of the complex and costly preparation methods of sulfonated monomers and the strict reaction conditions of the subsequent preparation of SPPQs. When appropriate sulfonation reagent is selected, SPPQs can also be obtained through the post-sulfonation method. Kopitzke et al. [16,17] synthesized SPPQ successfully by soaking PPQ in 50% fuming sulfuric acid before being baked in vacuum. The main-chain type SPPQs PEMs were synthesized in our laboratory previously, which showed excellent thermodynamic stability, chemical stability and dimensional stability. They showed lower proton conductivity because of an acid-base complex [21].

Sulfonic acid groups located on the side chain of the polymer keep a certain distance from the hydrophobic main chain, which can reduce the restriction of the rigid main chain on its activity. As a result, the proton conductivity of PEMs is enhanced. Wang et al. introduced a series of side-chain type sulfonated poly (aryl ether sulfone) (SPAES) by grafting four sulfonic groups onto the side chain. The proton conductivity of the membranes ranged between 146 and 365 mS cm^−1^ at 50–95% relative humidity (RH) and 80 °C, which is significantly higher than that of Nafion [23].

In this paper, a series of side-chain type SPPQs were prepared from novel monomers: 4,4′-bis (2-diphenyletherethylenedione) diphenyl ether (ODBZOBP), 4,4′-bis (2-phenylethylenedione) diphenyl ether (ODBZ) and 3,3′,4,4′-tetraaminobiphenyl (DAB). Additionally, the post-sulfonation method was applied to precisely control the location and degree of sulfonation. Ion exchange capacity (IEC), thermal and mechanical properties, water uptake and size change, oxidative stability, proton conductivity and DMFC performance were reported.

## 2. Materials and Methods

### 2.1. Materials

4-Fluorophenylacetyl chloride, phenyl ether, phenylacetyl chloride and Nafion were purchased from Adamas. 3,3′-Diaminobenzidine (DAB), N, N’-dimethylacetamide (DMAc), *m*-cresol and dimethyl sulfoxide (DMSO) were purchased from Macklin (Shanghai, China). 1-Methyl-2-pyrrolidinone (NMP), potassium hydroxide, anhydrous aluminum chloride and cupric bromide were received from Innochem (Beijing, China). Hydrogen peroxide (30%) was obtained from XiLong Scientific (Shanghai, China). Sulfuric acid (98%) and hydrochloric acid (37%) were purchased from Tian in Fuyu fine chemical Co., Ltd. (Tianjin, China) Gas diffusion electrodes (GDEs) were obtained from Sushui Energy Tech (shanghai) Co., Ltd. All chemicals were used as received.

### 2.2. Monomer Synthesis

**Figure 1:**
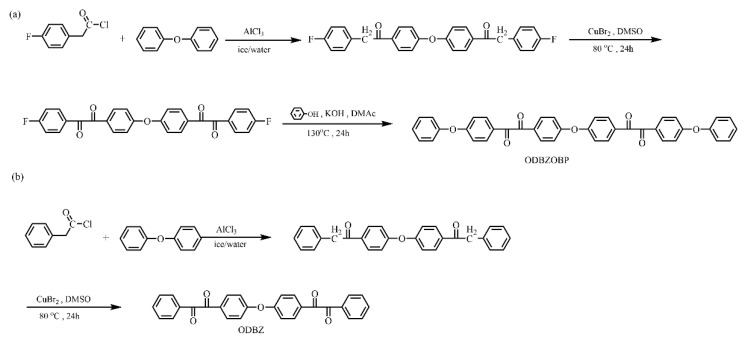
Synthesis of monomers ODBZOBP (**a**) and ODBZ (**b**).

Two diacyl monomers were obtained as shown in Figure 1. The specific synthesis processes of ODBZOBP were as follows.

Anhydrous aluminum chloride (17.20 g, 130 mmol) and phenyl ether (10.72 g, 63 mmol) were added to a 100 mL flask in an ice bath. In the next step, 4-fluorophenylacetyl chloride (22.43 g, 130 mmol) was added dropwise with a constant pressure funnel. After dripping and stirring for 16 h at room temperature, the reaction solution was slowly poured into 10% dilute hydrochloric acid, and the obtained material was washed for three times with deionized water until neutralized and then dried. Finally, 25.05 g 4,4′-bis (4-fluorophenone) diphenyl ether was obtained. Yield: 90%. ^1^H NMR spectrum (DMSO-*d*6; ppm): 4.40 (s, 2H), 7.18 (m, 4H), 7.30 (d, 2H), 8.14 (d, 2H).

In a 250 mL flask, 4,4′-bis (4-fluorophenone) diphenyl ether (22.10 g, 0.05 mol) and DMSO (120 mL) were added, stirred until completely dissolved and cupric bromide (22.34 g, 0.10 mol) was slowly added. Then the reaction mixture was heated to 80 °C and maintained 80 °C for 24 h. Yellow solid was precipitated by slowly pouring the solution into water, and 19.90 g 4,4′-bis (2-*p*-fluorophenyl ethylenediketone) diphenyl ether was obtained by recrystallizing from acetone and filtering. Yield: 86%. ^1^H NMR spectrum (DMSO-*d*6; ppm): 7.33 (d, 2H), 7.47 (d, 2H), 8.03 (m, 4H).

Under a nitrogen atmosphere, phenol (4 mL, 0.046 mol), potassium hydroxide (2.682 g, 0.048 mol) and methanol (25 mL) were successively added to a 100 mL three neck flask and reacted for 2 h. After the methanol had evaporated completely, 4,4′-bis (2-*p*-fluorophenyl ethylenediketone) diphenyl ether (10.01 g, 0.022 mol) and DMAc (42 mL) were added. The solution was slowly poured into the deionized water after reacting for 14 h at 140 °C and filtered to obtain the light yellow product. The monomer ODBZOBP was obtained by recrystallization from acetone. Yield: 86%. ^1^H NMR spectrum (DMSO-*d*6; ppm): 7.14 (d, 2H), 7.14 (d, 2H), 7.31 (m, 3H), 7.49 (m, 2H), 7.97 (m, 4H).

### 2.3. Polymer Synthesis

**Figure 2:**
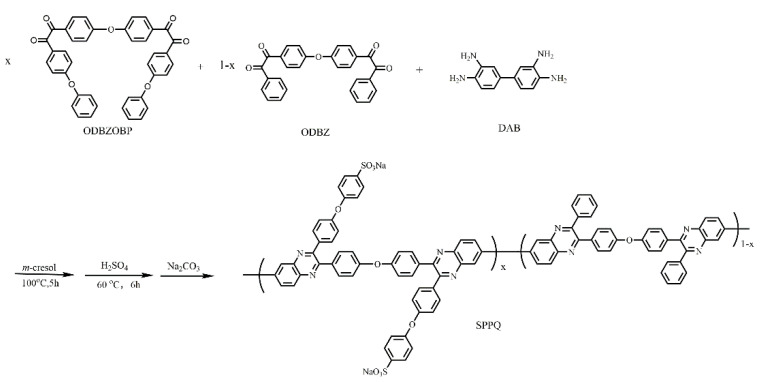
Synthesis of SPPQ.

Five kinds of side-chain type SPPQs with different sulfonation degree were prepared by controlling the molar ratio of ODBZOBP and ODBZ and the copolymerization with DAB and post-sulfonation with sulfuric acid under nitrogen conditions. The molar ratios of ODBZOBP to ODBZ in different polymers were 2:1, 3:1, 4:1, 5:1 and 1:0, respectively, and the corresponding polymers was named SPPQ-1, SPPQ-2, SPPQ-3, SPPQ-4 and SPPQ-5 after sulfonation. The synthesis method is shown in Figure 2. In the case of SPPQ-1, the synthetic procedures are as follows.

To a dry three-neck flask equipped with a magnetic stirrer under nitrogen, ODBZOBP (1.237 g, 2 mmol), ODBZ (0.434 g, 1 mmol) and DAB (0.643 g, 3 mmol) and *m*-cresol (23 mL) were added and stirred for 5 h at 100 °C. Subsequently, the viscous reaction solution was slowly poured into methanol (200 mL), and the yellow fibrous solid product was PPQ-1. The residual *m*-cresol in the PPQ was removed by washing with methanol three times. The product was dried for 6 h at 60 °C. The synthesized product PPQ-1 (2.00 g) was dissolved in sulfuric acid (20 mL), stirred, heated at 60 °C for 6 h, slowly poured into deionized water (200 mL) and repeatedly washed. Then the appropriate amount of sodium carbonate was added and stirred for 24 h. The yellow filamentous product was collected by filtration, washed with deionized water several times until the pH value was close to 7 and dried at 120 °C for 5 h. The yield was 90%.

### 2.4. Membrane Formation

The polymer solution was attained by dissolving side-chain type SPPQ with the salt form into DMSO to form about 7% solution. The solution was filtered, defoamed, and poured onto a smooth glass dish and dried at 80 °C for 24 h. In order to eliminate residual solvent, the membrane was soaked in methanol for 12 h. Then they were immersed into a 1 M hydrochloric acid aqueous solution for 48 h to ensure complete proton exchange and washed with deionized water until neutral. The proton form SPPQ PEM was obtained after drying at 60 °C for 4 h.

### 2.5. Measurements

The chemical structures of monomers and polymers were characterized by ^1^H NMR spectra and FTIR. ^1^H NMR spectra were recorded using an Avance IIIHD 400 MHz spectrometer with dimethyl sulfoxide-*d*6 (or chloroform-*d*) as the solvent and TMS as the internal standard. FTIR spectra were recorded using the total reflectance (ATR) mode on a NICOLET IS10 instrument. Thermogravimetric analysis (TG) was down on TG-DTA 6200 instrument heating at 10 °C min^−1^ in the nitrogen atmosphere (flow rate: 60 cm^3^ min^−1^). The intrinsic viscosity of the polymer was measured in DMSO at 30 °C with an Ubbelohde viscometer. Mechanical properties were uncovered with a TRC1150A universal testing machine under dry conditions at 20 °C at a crosshead speed of 2 mm min^−1^.

We measured ionic exchange capacity (IEC) using the titration method. Method 1: the accurately weighed protonated membrane was immersed into 15 wt% NaCl solution (50 mL) for 72 h and titrated with standard 20 mM NaOH aqueous using phenolphthalein as an indicator. Method 2: the accurately weighed membrane with proton form was immersed into 15 wt% NaCl solution (50 mL) for 72 h. Then the membrane was removed and thoroughly washed with deionized water. The combined solution of NaCl solution and washed water from the membrane was titrated by the standard 20 mM NaOH using phenolphthalein as an indicator. The experimental IEC was obtained from Equation (1)

(1) $$\mathrm{IEC}=\frac{V_{NaOH}C_{NaOH}}{W_{d}}$$

where $V_{NaOH}$ is the volume consumed, $C_{NaOH}$ is the concentration of the solution, and $W_{d}$ is the weight of the dry membrane.

Water uptake (WU) was measured by soaking the membrane with weight of about 100–200 mg per sheet into deionized water at 30 °C (or 80 °C) for 10 h (or 3 h). After that, the membrane was removed, quickly wiped with tissue paper and weighed with an analytical balance. The water uptake was calculated from Equation (2):

(2) $$\mathrm{WU}\left(\%\right)=\frac{W_{s}-W_{d}}{W_{d}}\times 100\%$$

where $W_{s}$ refers to the membrane weights when wet, while $W_{d}$ is the membrane weights when dry.

Size change in thickness ($\Delta t_{c}$) and in plane ($\Delta l_{c}$) direction was measured by immersing the PEMs into deionized water at 30 °C (or 80 °C) for 10 h (or 3 h). The size change in thickness and in plane direction was calculated from Equation (3):

(3) $$\Delta l_{c}\left(\%\right)=\frac{l-l_{d}}{l_{d}}\times 100\%\;\Delta t_{c}\left(\%\right)=\frac{t-t_{d}}{t_{d}}\times 100\%$$

where $l_{d}$ refers to length of the dry membranes and $t_{d}$ is their thickness. Similarly, l and t refer to the equilibrated membrane lengths and thicknesses.

The proton conductivity (*σ*) of PEMs was tested by an electrochemical impedance analyzer (Hioki 3536) over the frequency, ranging from 10 Hz to 100 KHz using the four-probe method. Two pairs of platinum plate electrodes with the sample membrane sandwiched in the middle was mounted as a cell on a Teflon plate at 0.5 cm distance. The cell was placed in water and tested from 30 to 90 °C. *σ* was calculated using Equation (4):

(4) $$\sigma =\frac{d}{t_{s}w_{s}R}$$

where *d* (cm) refers to the distance between the two electrodes, $w_{s}$ and $t_{s}$ refer to the width and thickness of the PEM in liquid water and *R* (Ω) is the resistance value of the sample.

Oxidative stability was tested by recording the time until membrane samples broke down in Fenton’s reagent of a solution containing 2 ppm FeSO_4_, 3% H_2_O_2_ at a given temperature (20 and 80 °C).

The prepared membrane electrode assembly (MEA) was positioned into an in-house fuel cell test station. Pt/Ru (30%)/C was used as anode catalysts and Pt/C was cathode catalysts, respectively. The catalyst of 1.0 mg Pt/Ru/cm^2^ was loaded on the anode, while 1.0 mg Pt/cm^2^ was loaded on the cathode. As a binder, 1 wt% Nafion solution was applied to both sides of the PEM surface. The MEA was prepared by hot-pressing the electrode/PEM/electrode sandwich for 5 min at 150 °C and 30 kgfcm^−2^, with an effective electrode area of 5 cm^2^.

## 3. Results and Discussion

### 3.1. Characterization of monomers

**Figure 3:**
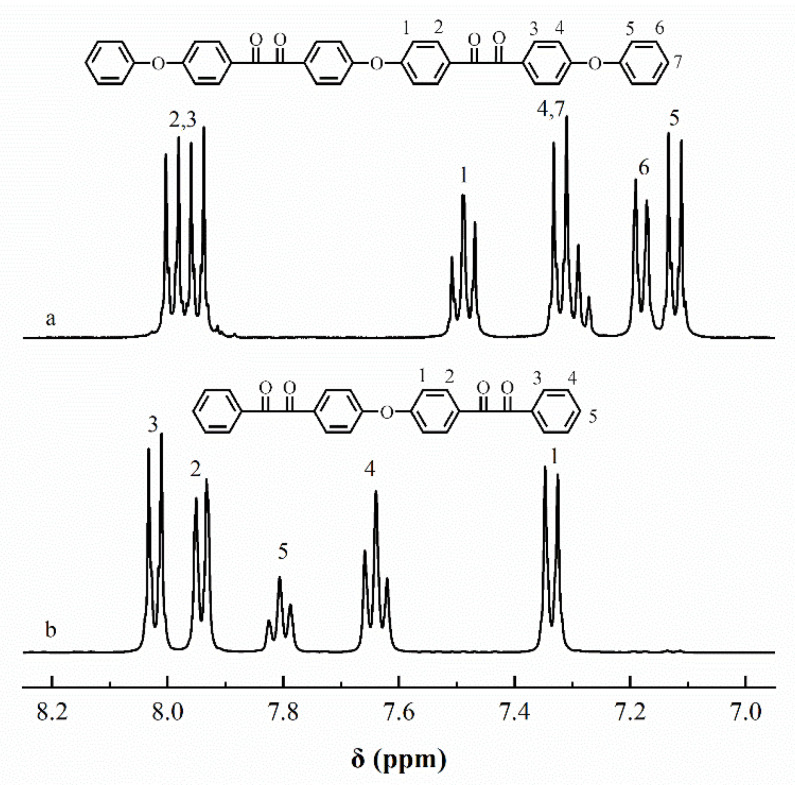
^1^H-NMR spectra of ODBZOBP (**a**) and ODBZ (**b**).

The chemical structures of ODBZOBP and ODBZ synthesized in this paper were described by ^1^H NMR spectra, as presented in Figure 3. The peaks in the spectra correspond to the corresponding different H positions in the chemical structures well and no impurity peaks were found. The ratio of peak area from different H positions was also consistent with that of the calculation. The target compounds with high purity were successfully synthesized.

### 3.2. Characterization of Polymers

**Figure 4:**
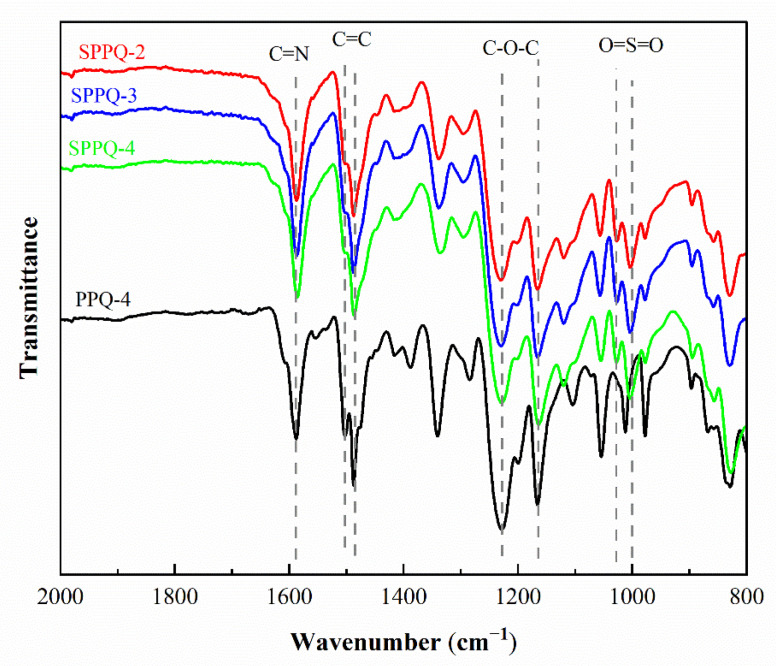
FTIR spectra of PPQ and SPPQs.

The chemical structures of PPQs before the post-sulfonation process and the side-chain type SPPQ PEMs were characterized by the FTIR spectra as presented in the Figure 4. The absorption band at 1595 cm^−1^ was assigned to the typical vibrations of the C=N group. The symmetric and asymmetric vibrations of the C-O-C bond appeared at 1229 cm^−1^ and 1166 cm^−1^, respectively [21]. The absorption bands at 1502 cm^−1^ and 1492 cm^−1^ correspond to the characteristic vibration absorption peaks of carbon skeleton on the benzene ring [24]. The symmetric and asymmetric vibrations of the O=S=O appeared at 1088 cm^−1^ and 1013 cm^−1^, showing that side-chain type SPPQs were prepared successfully [20]. Between 1900 to 1650 cm^−1^, no absorption bands were observed that occurred with the C=O stretching vibration, indicating a high degree of cyclization of C=O and -NH_2_ groups [25].

**Figure 5:**
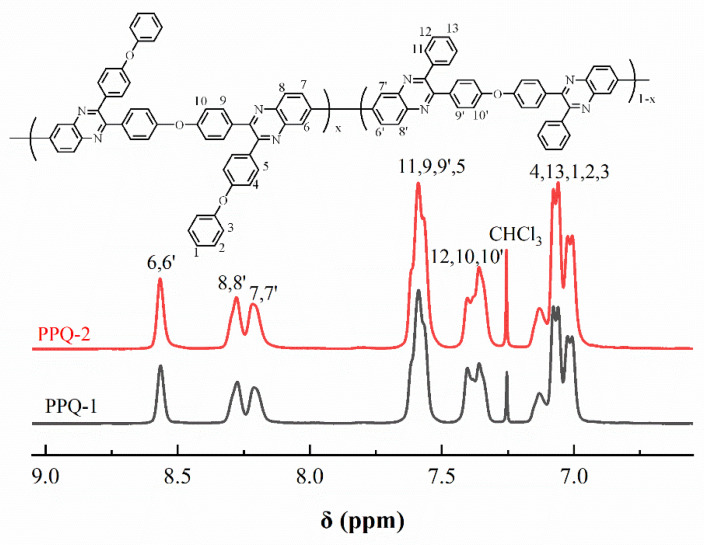
^1^H-NMR spectra of PPQs.

**Figure 6:**
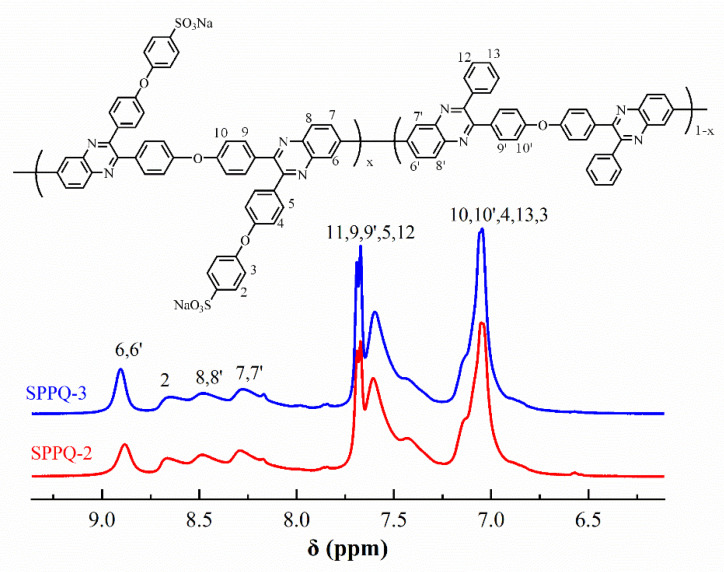
^1^H-NMR spectra of SPPQs.

Figure 5 illustrated the ^1^H NMR spectra of PPQs in chloroform-*d*. The peaks in the Figure 5 were well assigned to the resulting polymers, confirming the chemical structure were obtained successfully. Figure 6 showed the ^1^H NMR spectra of side-chain type SPPQs in dimethyl sulfoxide-*d*6. By comparing the ^1^H NMR spectra of side-chain type SPPQs with different IEC, the peak positions of the side-chain type SPPQs were basically same because of the same monomers resulting in the same chemical structure of polymers. The absorption peak was 8.65 ppm for the ortho-hydrogen of the sulfonic acid group on the benzene ring in the side chain when compared to the ^1^H-NMR spectra of monomers ODBZOBP and ODBZ. Their integration ratios matched the calculated values well for PEMs with different IEC values. It indicated that the sulfonic group had been successfully grafted onto the p-position of phenoxy groups in the side chain of PPQ by synthesized and well-designed monomers and convenient post-sulfonation processes.

The viscosity of the side-chain type SPPQ prepared in this paper was listed in Table 1. Their viscosity values are higher than 2.11 dL g^−1^, indicating the successful synthesis of high molecular weight copolymers, which was beneficial to reduce the hydrolysis of polymers at the end of the chain and effectively prevent membranes from degradation under cell conditions. Their solubility in common organic solvents was listed in Table 2. All side-chain type SPPQs and PPQs were soluble in NMP and *m*-cresol, insoluble in acetone. The SPPQs were soluble in DMSO and DMAc, while PPQs before sulfonation were insoluble in them.

### 3.3. Ionic Exchange Capacity

The theoretical IECs (IEC_Theo_) and experimental IECs (IEC_Titr_) determined by the titration of side-chain type SPPQ PEMs are listed in Table 1. The IEC_Titr1_, obtained from the titration method, demonstrated that the membrane sample kept in the water was in good accordance with that of IEC_Theo_ (more than 95% of the IEC_Theo_), which indicated the combination of structure-designing and the post-sulfonation method was suitable for the precise controllable preparation of side-chain type SPPQs. The values of IEC_Titr2_, on the other hand, measured by titrating the exchanged proton ionic in the solution, showed about 6% lower than that of IEC_Titr1_, which was mainly attributed to an acid-base interaction between the PPQ groups and the sulfonic acid groups, and a small part of the incomplete exchange of proton ions with SPAES, as described in the literature [26]. This phenomenon was also reported by Roziere et al. with base-doped N-benzylsulfonate-grafted polybenzimidazole (PBI) [27]. The lower basicity of PPQ groups than that of the benzimidazole groups resulted in the lower crosslinking degree of SPPQ.

### 3.4. Water Uptake and Size Change

Water uptake and size change are two important parameters with which to measure the performance of PEMs. High water absorption is beneficial for the transportation of protons in a PEM, while the over-absorption of water will lead to the excessive swelling of PEMs, which affects the forming stability of MEA and its performance. Therefore, PEMs need appropriate water uptake and size change to ensure the proper proton conductivity and good mechanical properties.

Water uptake, water molecules absorbed per sulfonic acid group (*λ*) and the dimensional change of side-chin type SPPQ PEMs are listed in Table 3. For comparison, the relevant data of m-SPPQ-5 and NR212 are also listed in the table.

The water uptake of side-chin type SPPQs increased in response to the increasing IEC and temperature as described in the literature [28]. At 30 °C, SPPQ-4 with the IEC of 1.93 meq g^−1^ showed a 36% water uptake, which was 1.1 times than 32% of SPPQ-2 and was 1.4 times than 26% of SPPQ-1 with the IEC of 1.65 meq g^−1^. As a comparison for SPAES, the water uptake of R2 (DFDPS-BP/DHDPS=1/1) with the IEC of 1.95 meq g^−1^ was 105%, more than 2.9 times the 36% of SPPQ-4 [26]. The low water uptake of SPPQ PEMs is mainly due to the interaction of quinoxaline and sulfonic acid groups on the polymer chain which improves performance. Meanwhile, SPPQ-1, SPPQ-2 and SPPQ-4 at 80 °C showed 29%, 34% and 39% water uptake, respectively, which were higher than that at 30 °C. When compared to the m-SPPQ-5 PEMs with similar IEC, the side-chain type SPPQ-5 showed higher water absorption, since they had sulfonic groups located in the side chain which were less affected by the main chain. For instance, the side-chain type SPPQ-5 showed 45% water uptake, while m-SPPQ-5 displayed 35%.

*λ* is an important index with which to measure the water content in the membranes, which is closely correlated with the absorption of water. The values of all side-chain type SPPQ PEMs increased slightly with the increase of IEC and temperature as shown in Table 3. As the temperature increased from 30 to 80 °C, the λ of SPPQ-5 increased from 11 to 13. The λ of 11 for side-chain type SPPQ-5 was higher than the 8.8 of the main-chain type m-SPPQ-5 and much lower than 23 of NR212 at 30 °C.

The degree of size change for side-chain type SPPQ PEMs increased with increasing IEC and temperature. The size change in the plane direction of SPPQs was in the range of 1.9–4.7% at 30 °C and in the range of 2.7–7.0% at 80 °C. The size change in the plane direction of side-chain type SPPQ-5 of 4.7% was significantly lower than the 6.9% of m-SPPQ-5 and the 13% of NR212, which was beneficial for the stability of MEA from peeling between the membrane and electrode due to their different swelling degree during the operation. The side-chain type SPPQ PEMs showed obvious anisotropic behavior when the swelling thickness direction was about three times higher than that of the in-plane direction, as shown in the Table 3. This was different from the main-chain type and NR212, which showed a similar swelling degree in the in-plane and thickness direction. On the other hand, the SPPQ-5 with the highest IEC of 2.17 meq g^−1^ remained intact even when immersed in boiling water for 12 h.

### 3.5. Thermal Properties, Mechanical Properties and Oxidative Stability

**Figure 7:**
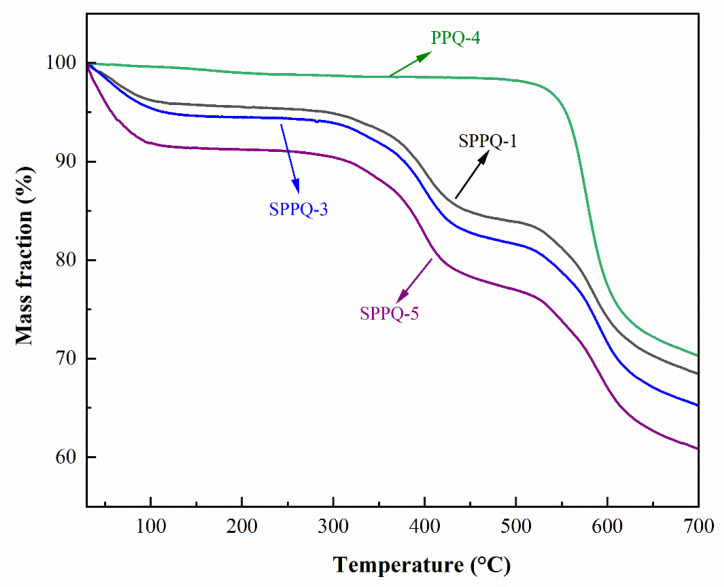
TGA curves of PPQ and SPPQs.

The thermal stability of side-chain type PPQ and SPPQ PEMs was the characterization with TGA shown in Figure 7. The thermal decomposition of PPQ did not occur until 550 °C, indicating the excellent thermal stability. The thermogravimetric curve of SPPQs showed three weight loss stages: (1) loss of water molecules adsorbed in the membrane at around 100 °C, (2) shedding of sulfonic acid groups on the polymer at about 325 °C and (3) the degradation of the polymer backbone above 550 °C. As a result, the SPPQ PEMs are thermally stable and reach the requirements of PEMs.

Good mechanical properties facilitate fuel cell system assembly and maintain good impact resistance during the cell operation. Young’s modules, maximum stress and Elongation at the break of side-chain type SPPQ PEMs were listed in Table 4. It was found that the Young’s modulus of these SPPQ PEMs were higher than 1.73 GPa, the maximum stress were higher than 47 MPa and the elongations at break were above 16%, even under dry conditions, showing the good mechanical properties needed for fuel cell application. Young’s modulus decreased with the increasing IEC. For example, Young’s modulus of SPPQ-2 with an IEC of 1.80 meq g^−1^ was 1.95 GPa, which decreased to 1.73 GPa for SPPQ-5 with IEC of 2.17 meq g^−1^.

The oxidative stability of PEM was studied by immersing the side-chain type SPPQ PEM into Fenton’s reagent at 20 °C and 80 °C, respectively, and was characterized by the time the membranes began to break up in Fenton’s reagent. The testing results were shown in the Table 4. The breaking time of the side-chain type SPPQ PEMs shortened with the increase in IEC, which means the stability became worse. For example, the breaking time of SPPQ-2 with IEC of 1.80 meq g^−1^ was 96 h at 20 °C, while that of SPPQ-5 (2.17 meq g^−1^) was 81 h at 20 °C. The breaking time of SPPQ-5 was 138 min at 80 °C, demonstrating that the temperature on the oxidative stability is not unaffected. The side-chain type SPPQ-5 exhibited similar oxidative stability to the main-chain type SPPQ but was inferior to that of NR212.

### 3.6. Proton Conductivity

Proton conductivity (*σ*) is used to characterize the ability of PEMs to conduct H^+^ and it is one of the most important properties to measure the performance of PEMs. The *σ* measured under the fully hydrated side-chain type SPPQs, m-SPPQ and NR 212 PEMs at different temperatures were listed in Table 4. The proton conductivity of PEMs increased with IEC. For example, the *σ* of SPPQ increased from 19 to 40 mS cm^−1^ as IEC increased from 1.80 meq g^−1^ of SPPQ-2 to 2.17 meq g^−1^ of SPPQ-5 at 30 °C. We attributed this greater proton conductivity at a higher IEC to the increased density of sulfonic acid group, which was conducive to conducting protons. As a result, the activation energy of PEM gradually decreased from 12.1 kJ mol^−1^ for SPPQ-2 to 10.6 kJ mol^−1^ for SPPQ-5.

**Figure 8:**
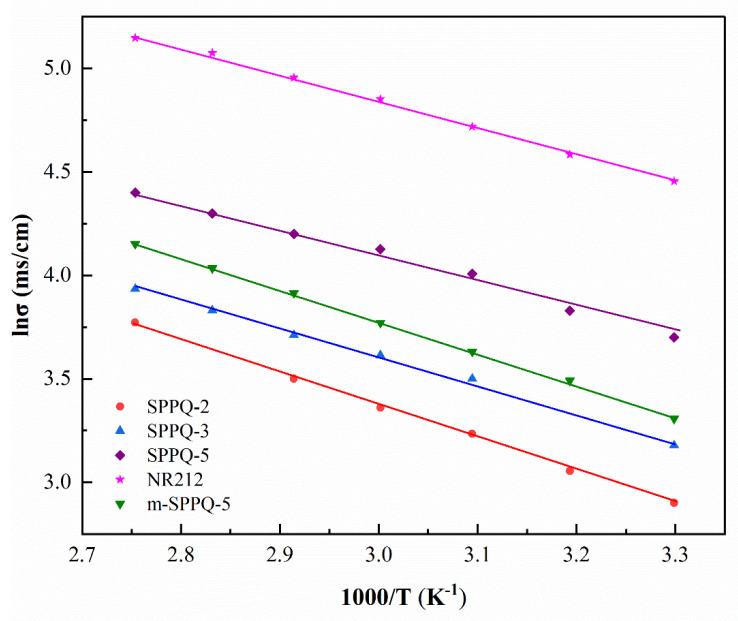
Temperature dependence of proton conductivity of membranes.

The temperature dependence of the proton conductivities of membranes was shown in Figure 8. From Table 4 and Figure 8, it can be seen that the proton conductivities of all PEMs increased with the increasing temperature. For example, the *σ* of SPPQ-5 was 62 mS cm^−1^ at 60 °C, while 76 mS cm^−1^ at 80 °C. It is worth noting that the proton conductivity of SPPQ-5 was higher than that of m-SPPQ-5 with similar IEC at all test temperatures. For instance, the *σ* in water of side-chain type SPPQ-5 was 76 mS cm^−1^ at 80 °C, which is obviously higher than the 64 mS cm^−1^ of m-SPPQ-5, proving that the proton conductivity of the modified side-chain type SPPQ PEMs was significantly improved. Because of the interaction of sulfonic acid and quinoxaline, the proton conductivity of SPPQ was lower than that of NR212, but it was qualified for the DMFC applications.

### 3.7. DMFC Performance

**Figure 9:**
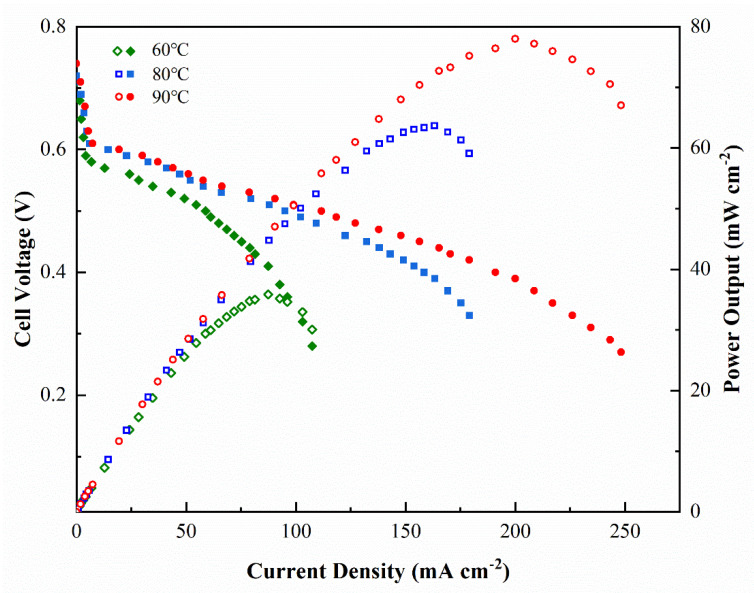
Effects of cell temperature on DMFC performance for SPPQ-5 with O_2_ supply without humidification and press and a 5 wt% methanol feed concentration.

The effect of temperature on the performance of DMFC was studied under the conditions of 5 wt% methanol solution at 2 mL min^−1^ flow rate on anode and O_2_ supply without humification and press at 150 mL min^−1^ on cathode under 60 °C to 90 °C. The results of the polarization curve and power output dependence on current density are shown in Figure 9. The performance of DMFC depended greatly on the temperature [29]. Take the sample SPPQ-5 as example, the open circuit voltage (OCV) was 0.71 V at 60 °C and increased to 0.74 V at 90 °C, indicating the oxidation reactivity was increased by temperature and the methanol crossover was barely increased through the SPPQ-5 PEM. The maximum power output (*W*_max_) was 35.8 mW cm^−2^ at 60 °C and was greatly increased to 78.0 mW cm^−2^ at 90 °C due to the increase of the activity of the catalyst at higher temperatures and the reduced ohmic polarization of PEM. As a result, the current density increased from about 89 mA cm^−2^ to 191 mA cm^−2^ at 0.4 V when the temperature increased from 60 °C to 90 °C.

**Figure 10:**
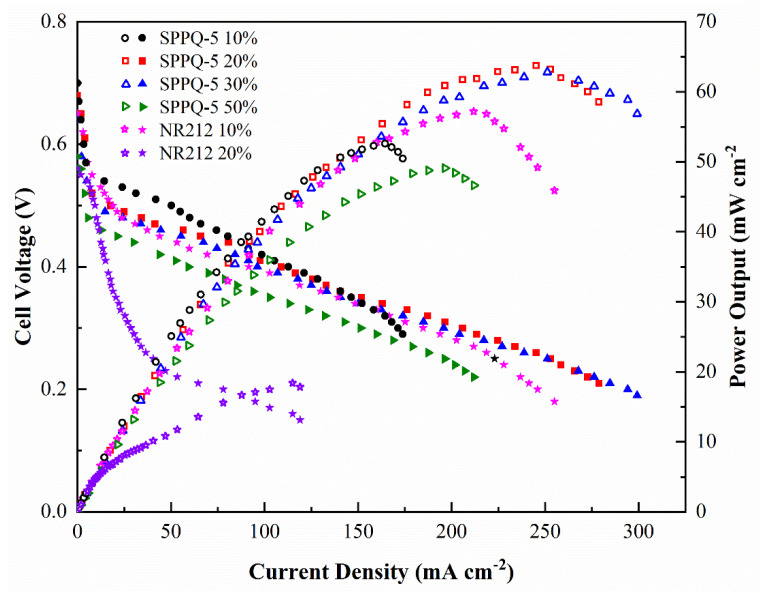
Effects of methanol feed concentration on DMFC performance with a supply of O_2_ but without humidification and press at 60 °C.

Methanol concentration has a large impact on the performance of fuel cells. In general, high methanol concentration supplies in the anode results in low cell performance because of the serious crossover of methanol [30]. The methanol concentration-dependent DMFC performance experiments in this paper were carried out under conditions of 10 wt%, 20 wt%, 30 wt% and 50 wt% methanol solution at 2 mL min^−1^ and O_2_ without humification and pressed at 150 mL min^−1^ at 60 °C, respectively. The results were shown in Figure 10. The *W*_max_ of SPPQ-5 increased from 52.6 mW cm^−2^ at 10 wt% methanol solution to 63.8 mW cm^−2^ at 20 wt% methanol solution, the OCV decreased from 0.69 V to 0.68 V. The cell performance decreased with a further increasing methanol concentration from 30% to 50%. For example, the OCV decreased from 0.68 V to 0.58 V and the *W*_max_ decreased from 63.8 mW cm^−2^ to 49.1 mW cm^−2^ when the methanol concentration increased from 20 wt% to 50 wt%. Compared to NR212, the OCV decreased from 0.65 V to 0.56 V, the *W*_max_ decreased from 57.2 mW cm^−2^ at 10% methanol solution to 18.4 mW cm^−2^ at 20 wt% methanol solution and lost its performance ability at 30 wt% of methanol concentration. The results showed that the side-chain type SPPQ PEMs obviously have a better methanol tolerance than that of NR212, which was a potential material for DMFC application.

## 4. Conclusions

In this paper, a series of side-chain type SPPQs with different IECs were prepared under mild conditions by combining monomer molecular-designing and post-sulfonation method. Their PEMs showed excellent stability and higher water uptake than main-chain type SPPQ PEMs. As expected, proton conductivity was improved to a reasonably high degree for DMFC by introducing sulfonic acid group onto the side chain of the PPQ. In the single cell test, the MEA prepared by SPPQ-5 showed good fuel cell performance, with cell temperature ranging from 60 °C to 90 °C. The *W*_max_ was 78.0 mW cm^−2^ under the condition of 5 wt% methanol solution and O_2_ without humidification at 90 °C. The SPPQ prepared in this paper showed excellent methanol tolerance with the methanol concentration changing from 5 wt% to 30 wt%, and the *W*_max_ was 63.8 mW cm^−2^ at 60 °C, which is largely higher than 18.4 mW cm^−2^ of NR212 under the same conditions. Considering the properties of side-chain type SPPQ-based PEMs, this material is expected to be a potential material for a proton exchange membrane fuel cell.

## Figures and Tables

**Table 1:** IEC and viscosity of SPPQs.

Code	DAB-Based Copolymer	IEC (meq g^−1^)			*ƞ*_r_ (dL/g)
		Theo ^1^	Titr1 ^2^	Titr2 ^2^	
SPPQ-1	ODBZOBP/ODBZ (2/1)	1.65	1.62	1.47	2.43
SPPQ-2	ODBZOBP/ODBZ (3/1)	1.80	1.71	1.60	2.55
SPPQ-3	ODBZOBP/ODBZ (4/1)	1.88	1.84	1.72	2.11
SPPQ-4	ODBZOBP/ODBZ (5/1)	1.93	1.90	1.78	2.12
SPPQ-5	ODBZOBP (homo)	2.17	2.13	2.00	2.14
m-SPPQ-5	BZOBP (homo)	2.21	2.06	1.87	3.35

^1^ Theoretical value; ^2^ Titration value.

**Table 2:** Solubility behaviors of SPPQs copolymers.

Code	Solvents					
	*m*-cresol	NMP	DMSO	DMAc	CHCl_3_	Acetone
PPQ-1	++	++	--	--	++	--
SPPQ-1	++	++	++	++	--	--
SPPQ-2	++	++	++	++	--	--
SPPQ-3	++	++	++	++	--	--
SPPQ-4	++	++	++	++	--	--
SPPQ-5	++	++	++	++	--	--

++ Soluble at room temperature; -- Insoluble when heated to 50 °C.

**Table 3:** Water uptake and size change of SPPQ PEMs.

Code	IEC (meq g^−1^)	Water Uptake (%)		Dimensional Change (%)						λ	
		30 °C	80 °C	30 °C			80 °C			30 °C	80 °C
				∆*l*c	∆*t*c	Δ*t*c/Δ*l*c	∆*l*c	∆*t*c	Δ*t*c/Δ*l*c		
**SPPQ-1**	1.65	26	29	1.9	7.7	4.1	2.7	11.1	3.8	9	10
SPPQ-2	1.80	32	34	2.5	9.1	3.6	4.0	13.3	3.3	10	11
SPPQ-3	1.88	33	36	3.1	11.1	3.5	4.7	14.3	3.0	10	11
SPPQ-4	1.93	36	39	3.5	12	3.4	5.0	15.8	3.2	10	11
SPPQ-5	2.17	45	52	4.7	12.5	2.7	7.0	17.5	2.5	11	13
m-SPPQ-5	2.21	35	39	6.9	9.6	1.4	11	13	1.2	8.8	9.9
NR212	0.91	37	44	13	16	1.2	17	20	1.2	23	27

**Table 4:** Mechanical properties, oxidative stability and the proton conductivity of membranes.

Code	Mechanical Properties			Oxidative Stability		Proton Conductivity (mS cm^−1^)			*E*a (kJ mol^−1^)
	*Y*^1^ (GPa)	*S*^2^ (MPa)	*E*^3^ (%)	20 °C (h)	80 °C (min)	30 °C	60 °C	80 °C	
SPPQ-2	1.95	55	22	96	148	19	29	34	12.1
SPPQ-3	1.86	51	22	90	145	24	37	46	11.4
SPPQ-4	1.81	47	18	89	140	29	47	56	11.3
SPPQ-5	1.73	57	16	80	120	40	62	76	10.6
m-SPPQ-5	1.60	50	20	81	138	34	54	64	15.1
NR 212	0.2	14	325	>300	>300	86	128	160	10.8

^1^ Young’s modules; ^2^ Maximum stress; ^3^ Elongation at break.

## Data Availability

Not applicable.

## Patents

There was one patent resulting from the work reported in this paper (number: CN201910897178.3).
